# Combination therapies utilizing neoepitope-targeted vaccines

**DOI:** 10.1007/s00262-020-02729-y

**Published:** 2020-10-08

**Authors:** Karin L. Lee, Jeffrey Schlom, Duane H. Hamilton

**Affiliations:** Laboratory of Tumor Immunology and Biology, Center for Cancer Research, National Cancer Institute, National Institutes of Health, Bethesda, MD USA

**Keywords:** Neoepitope vaccine, Combination therapy, Checkpoint blockade, Epitope spreading

## Abstract

Clinical successes have been achieved with checkpoint blockade therapy, which facilitates the function of T cells recognizing tumor-specific mutations known as neoepitopes. It is a reasonable hypothesis that therapeutic cancer vaccines targeting neoepitopes uniquely expressed by a patient’s tumor would prove to be an effective therapeutic strategy. With the advent of high-throughput next generation sequencing, it is now possible to rapidly identify these tumor-specific mutations and produce therapeutic vaccines targeting these patient-specific neoepitopes. However, initial reports suggest that when used as a monotherapy, neoepitope-targeted vaccines are not always sufficient to induce clinical responses in some patients. Therefore, research has now turned to investigating neoepitope vaccines in combination with other cancer therapies, both immune and non-immune, to improve their clinical efficacies.

## Introduction

In the past decade, immunotherapy has come to the forefront of cancer treatment. In particular, immune checkpoint blockade has shown remarkable efficacy in a subset of patients, particularly those with high tumor mutational burden [1]. It is hypothesized that these responses are a result of activating T cells within the tumor that are capable of targeting these neoepitopes. Although the majority of neoepitopes are unique to each patient’s tumor, there are shared neoepitopes, which are present in a large subset of patients. These common mutations are often in oncogenes or tumor-suppressor genes, including those found in epidermal growth factor receptor class III variant (EGFRvIII), mutant isocitrate dehydrogenase 1 (IDH1), RAS, BRAF, and TP53 [2–4]. Preclinical and clinical studies have shown that vaccines targeting these shared neoepitopes induce antigen-specific immunity and improve survival [3–6]. However, these shared neoepitopes are often tumor-type specific, and offer very few potential targets. Personalized vaccines targeting neoepitopes uniquely expressed by a patient’s tumor could vastly expand the repertoire of potential targets, allowing for the generation of a more diverse immune response.

Talimogene laherparepvec (T-VEC) is an FDA-approved oncolytic virus for treatment of advanced melanoma. T-VEC is a modified oncolytic virus that is injected directly into tumor lesions. It is thought that the virus selectively replicates in cancer cells, while also producing granulocyte macrophage-colony stimulating factor (GM-CSF). The virus lyses cancer cells, releasing potential tumor antigens, including neoepitopes that can serve to activate and expand tumor-specific immune cells [7]. While effective in melanoma, in which individual metastatic skin lesions can be easily injected with the virus, this is often not possible for other cancer types in which neither the primary nor metastatic lesions are accessible. With the advent of high-throughput genomic sequencing, it is now possible to identify and specifically target personal neoepitopes clinically with patient-specific therapeutic cancer vaccines. These vaccines aim to expand and enrich the pool of high-affinity tumor-specific T cells capable of controlling tumor growth and neoepitope-targeted vaccines are being evaluated in clinical studies [8–10]. This review will discuss identification, selection, and delivery of neoepitope-targeted vaccines, as well as lessons learned from preclinical and clinical studies utilizing monotherapy neoepitope vaccines, with an emphasis on the importance and potential of combining such vaccines with other cancer therapies to improve clinical efficacies.

### Identification, selection, and delivery of neoepitopes

Tumor-specific mutations are ideal immunological targets for the treatment of cancer because they represent a pool of antigens, found exclusively in tumors that are capable of being recognized by T cells. Additionally, because neoepitopes are seen as “foreign” by the immune system, reactive T cells are not subject to negative selection [11, 12]. Thus, neoepitope-reactive T cells represent a potential pool of high-affinity T cells capable of efficiently killing tumor cells. In spite of this promise, there remain a number of technical challenges in both the identification and selection of neoepitopes and the manufacturing of personalized vaccines.

Potential neoepitopes are detected using whole-exome next generation sequencing (NGS) by comparing tumor tissue with a matched healthy tissue sample to identify mutations uniquely expressed by the tumor. Non-synonymous mutations, i.e., those that result in an amino acid change in tumor cells versus normal cells, are the most common mutations that lead to potential neoepitopes, but targetable mutations may also arise from insertions and deletions or frameshift-mutations [11]. Once mutations are identified, their expression levels in the tumor are determined via RNA-seq [10]. However, even if a mutation is expressed, there is no guarantee that it will form a neoepitope that is recognized by T cells. Thus, it is necessary to consider which mutated peptides will be processed and presented correctly on HLA molecules to be recognized by T cells [13]. Therefore, the ideal neoepitope vaccination candidate should arise from a tumor-specific mutation and result in a peptide that is processed and presented on HLA molecules. The resulting peptide-HLA complex must then be recognized by T cells to induce an immune response. While the majority of studies utilize whole exome sequencing to identify neoepitopes, it is difficult to correlate this information with the number of actual neoantigens. In most clinical trial data reported, patients were vaccinated only with a subset of the identified neoepitopes, and subsequently monitored only for reactivity against those peptides incorporated into the vaccine. Therefore, based on published data, it is difficult to extrapolate whether the reactivities against vaccine peptides are a good representation of those that would be seen against all predicted epitopes.

A common method for ranking the potential antigenicity of neoepitopes is the use of HLA binding algorithms [11]. These algorithms use neural networks to predict which epitopes will bind different HLA alleles. However, while HLA class I software prediction is widely available and has been shown to enrich for potential neoepitopes, development of accurate HLA class II prediction algorithms remains a challenge [11]. Additionally, predicted binding affinities do not always correlate with actual binding affinities, nor are high binders necessarily the most immunogenic neoepitopes. Other methods of neoepitope selection include identification of presented neoepitopes via mass spectrometry following elution from HLA [14] or identification of pre-existing T-cell responses by incubating patient peripheral blood mononuclear cells with antigen-presenting cells (APCs) pulsed with neoepitope candidate peptides [15] or transfected with tandem mini genes [16]. However, these methods are not high-throughput or extremely efficient. Additional discussion of the advantages and disadvantages of each selection method has been previously reported [17].

Once neoepitopes have been identified and appropriate candidates selected, vaccines must be manufactured quickly. There are a variety of delivery methods being considered, which include synthetic peptides with adjuvants, RNA and DNA vaccines, viral vectors, bacterial vectors, nanoparticle delivery, and ex vivo–loaded dendritic cells (DCs). The advantages and disadvantages of each vaccine format have been discussed previously [11]. Other aspects of vaccine delivery to consider are the choice of adjuvant to enhance immune responses to vaccine peptides, as well as combining monotherapy vaccines with additional cancer therapies to further improve efficacy.

### Neoepitopes as monotherapy vaccines

Early preclinical studies established the promise of targeting neoepitopes using vaccines as a monotherapy. In syngeneic murine models of glioma, melanoma, sarcoma, and colorectal cancer, neoepitope vaccines (delivered with various adjuvants) induced robust antigen-specific IFNγ responses in CD4 and CD8 T cells. This resulted in decreased tumor growth in both the prophylactic and therapeutic vaccination settings [14, 18–22]. Tumor growth control was associated with increased infiltration of anti-tumor immune cells and decreased suppressive cells in the tumor microenvironment [14, 19]. Interestingly, while treatment with peptide and RNA-based vaccines led to predominantly T-cell–mediated responses, altering delivery and presentation of neoepitopes could also induce B-cell responses. Mice vaccinated with neoepitopes delivered via T7 phages or fused to the membrane translocation domain of diphtheria toxin increased frequencies of B cells and generated robust antibody responses, including increased plasma levels of IgG and poly-reactive IgM [21, 23].

Due to encouraging preclinical results with monotherapy neoepitope vaccines, interest has increased in moving these vaccines to the clinic. Additionally, clinical translation of personalized vaccines has become more feasible as methods for neoepitope identification have improved. There are currently approximately 100 clinical trials utilizing neoepitope vaccines. Initial trials showed that neoepitope vaccination, delivered with adjuvants and typically with standard-of-care chemotherapy, induced de novo neoepitope-specific T-cell immunity to vaccine components [8–10, 24, 25]. Most reactive T cells were CD4, regardless of whether class I peptides or long peptides were used [8–10, 25]. One study showed that these neoepitope-specific CD4 T cells secreted IFNγ, IL-2, and tumor necrosis factor (TNF), both singly and in combination, while another study showed that CD4 cells displayed high levels of activation markers (CD25, CD69, HLA-DR) and higher mRNA levels of Granzyme B, perforin, and CRTAM after stimulation with neoepitope peptides, indicating activation of antigen-specific T cells [8, 25]. Additionally, it was confirmed that immunogenic neoepitopes in the vaccines were being processed and presented [9, 24]. However, despite the development of strong immune responses following vaccination, only a subset of patients had a reported clinical benefit such as decreased rate of metastasis; however, no controlled studies have been performed to clearly demonstrate a positive impact on overall survival [8–10]. Studies showed that reactive T cells expanded following vaccination often expressed programmed death-1 (PD-1), indicating an exhausted phenotype [10]. Thus, patients may likely benefit from combining these vaccines with other therapies.

### Neoepitopes in combination therapies

**Fig. 1:**
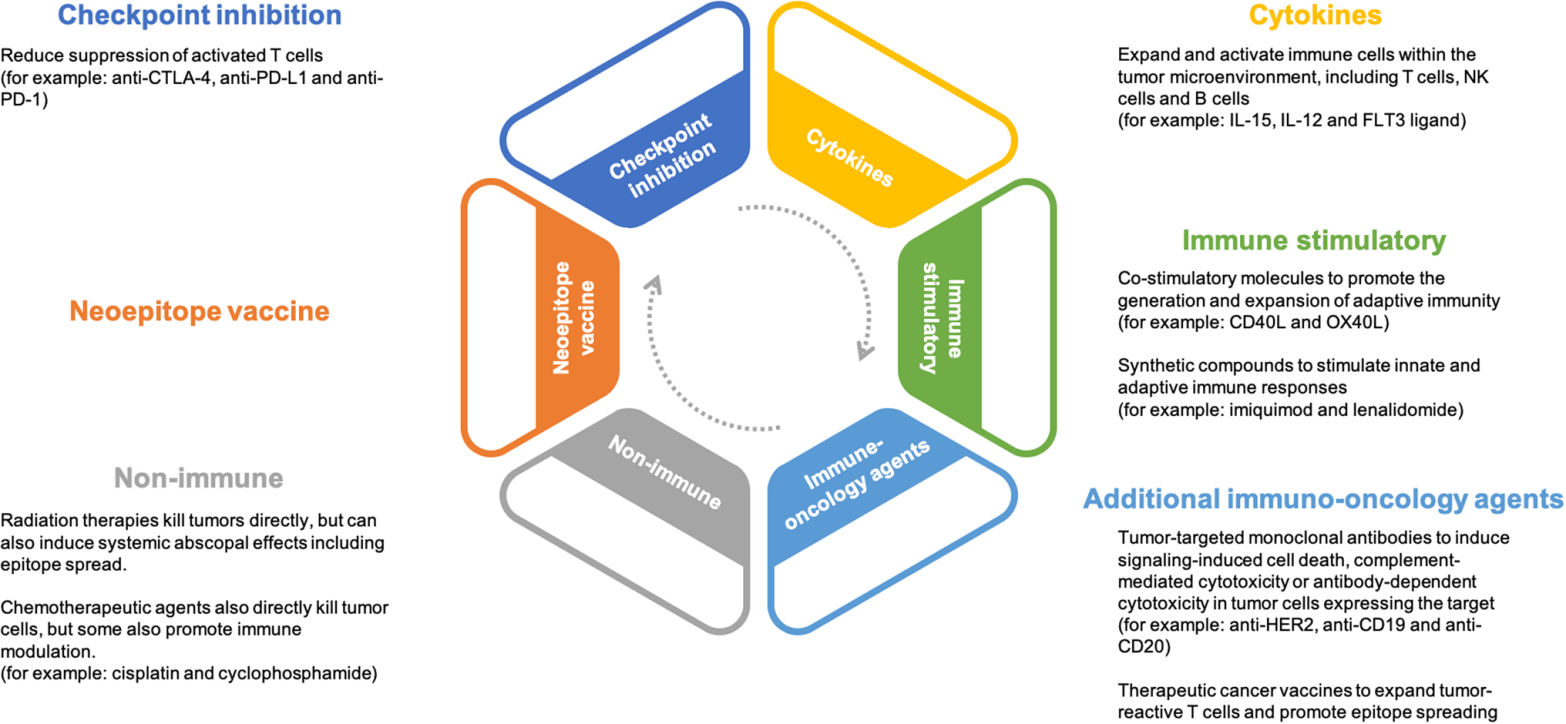
Targeting the tumor microenvironment using neoepitope vaccine in combination with rationally selected cancer therapies

Numerous preclinical studies have investigated combining tumor-associated antigen (TAA)–targeting vaccines with other cancer therapies [26]. These combinations aim to increase, expand, and activate vaccine reactive T cells present in the tumor microenvironment by countering multiple mechanisms of immune failure, including the presence of immune-suppressive cells [27], upregulation of immune checkpoints [18, 28], and an immunosuppressive tumor microenvironment [29]. Additionally, it is important to rationally select agents for combination and to ensure that agents do not antagonize each other. Due to promising results from monotherapy vaccine studies targeting neoepitopes, recent preclinical studies investigated combining neoepitope vaccines with other cancer therapies, including checkpoint inhibitors [18, 20, 28, 30–33], other immuno-oncology agents [18, 34], and “non-immune” therapies [35] to improve their efficacies. Additionally, numerous clinical trials, summarized in Table 1, have been proposed to investigate neoepitope vaccination in combination with checkpoint inhibitors, other immuno-oncology agents, and “non-immune” cancer therapies. Each of these agents can potentially enhance the therapeutic efficacy of a neoepitope vaccine via different mechanisms (Fig. 1).

**Table 1:** Clinical trials combining neoepitope vaccines with cancer therapies

Combination with checkpoint inhibitors						
Cancer Type	Vaccine Formulation	Combination	Combination Class	Trial Number	Phase	Status
Kidney cancer	NeoVax (peptides + poly-ICLC)	Ipilimumab	Checkpoint inhibitor (CTLA-4)	NCT02950766	I	Recruiting
Squamous cell lung cancer, squamous NSCLC, squamous cell carcinoma of head and neck	PANDA-VAC (6 peptides + poly-ICLC)	Nivolumab	Checkpoint inhibitor (PD-1)	NCT04266730	I	Not yet recruiting
Bladder cancer, melanoma, lung cancer	NEO-PV-01 (peptide) + poly-ICLC	Nivolumab	Checkpoint inhibitor (PD-1)	NCT02897765	I	Active, not recruiting
Ovarian cancer	NeoVax (peptides + poly-ICLC)	Nivolumab	Checkpoint inhibitor (PD-1)	NCT04024878	I	Not yet recruiting
Melanoma	NeoVax (peptides + poly-ICLC) + Montanide	Nivolumab, Ipilimumab	Checkpoint inhibitor (PD-1, CTLA-4)	NCT03929029	Ib	not yet recruiting
Glioblastoma	NeoVax (peptides + poly-ICLC)	Nivolumab, Ipilimumab	Checkpoint inhibitor (PD-1, CTLA-4)	NCT03422094	I	Suspended (drugs/equipment not currently available)
NSCLC, colorectal, pancreatic, solid tumor, shared neoantigen-positive solid tumors	Peptide [GRT-C903 (shared neoantigen prime) + GRT-R904 (shared neoantigen boost)]	Nivolumab, Ipilimumab	Checkpoint inhibitor (PD-1, CTLA-4)	NCT03953235	I/II	Recruiting
NSCLC, colorectal, urothelial carcinoma, gastroesophageal adenocarcinoma	GRT-C901 (Chimpanzee Adenovirus [prime]) + mRNA-based (self-amplifying mRNA in lipid nanoparticle [boost])	Nivolumab, Ipilimumab	Checkpoint inhibitor (PD-1, CTLA-4)	NCT03639714	I/II	Recruiting
Advanced cancer	Personalized cancer vaccine	Pembrolizumab	Checkpoint inhibitor (PD-1)	NCT03568058	Ib	Recruiting
Cutaneous melanoma, NSCLC, SCC of head and neck, urothelial, RCC	GEN-009 adjuvanted vaccine (synthetic long peptides)	Nivolumab, Pembrolizumab	Checkpoint inhibitor (PD-1)	NCT03633110	I/II	Recruiting
Urothelial/bladder cancer	PGV001 (long peptides + tetanus helper peptide)/poly-ICLC	Atezolizumab	Checkpoint inhibitor (PD-L1)	NCT03359239	I	Recruiting
Locally advanced or metastatic tumors	RO7198457 (mRNA-based)	Atezolizumab	Checkpoint inhibitor (PD-L1)	NCT03289962	I	Recruiting
TNBC	DNA vaccine (TDS-IM system)	Durvalumab	Checkpoint inhibitor (PD-L1)	NCT03199040	I	Recruiting
Renal cell carcinoma	DNA vaccine (TDS-IM system)	Durvalumab, Tremelimumab	Checkpoint inhibitor (PD-L1, CTLA-4)	NCT03598816	II	Not yet recruiting

Combination with immuno-oncology agents						
Cancer Type	Vaccine Formulation	Combination	Combination Class	Trial Number	Phase	Status
Metastatic melanoma	NEO-PV-01 (peptide) + poly-ICLC	Nivolumab, Ipilimumab, APX005M	Checkpoint inhibitor (PD-1, CTLA-4), immune-stimulatory (CD40 agonist)	NCT03597282	I	Active, not recruiting
Pancreatic and colorectal cancer (advanced)	Peptide	Pembrolizumab, Imiquimod	Checkpoint inhibitor (PD-1), immune response modifier	NCT02600949	I	Active, not recruiting
Glioblastoma	DNA vaccine (CELLECTRA®2000 EP Device)	Pembrolizmuab, INO-9012 (plasmid encoded IL-12)	Checkpoint inhibitor (PD-1), cytokine	NCT04015700	I	Not yet recruiting
HCC	GNOS-PV02 (DNA vaccine)	Pembrolizumab, INO-9012 (plasmid encoded IL-12)	Checkpoint inhibitor (PD-1), cytokine	NCT04251117	I/IIa	Recruiting
Metastatic hormone-sensitive prostate cancer	DNA vaccine (TriGrid Delivery System)	PROSTVAC-V, PROSTVAC-F, Nivolumab, Ipilimumab	TAA vaccine, checkpoint inhibitor (PD-1, CTLA-4)	NCT03532217	I	Recruiting
Follicular lymphoma	NeoVax (peptides + poly-ICLC)	Rituximab	mAB (CD20)	NCT03361852	I	Not yet recruiting
Solid tumors	PGV001 (peptides + poly-ICLC)	Lenalidomide	Immunomodulatory drug	NCT02721043	I	Active, not recruiting
Stage IIB-IV melanoma	NeoVax (peptides + poly-ICLC)	CDX-1401 (DEC-205/NY-ESO-1 fusion protein), CDX-301 (recombinant Flt3 ligand)	TAA vaccine, cytokine	NCT02129075	II	Active, not recruiting

Combination with non-immune therapies						
Cancer Type	Vaccine Formulation	Combination	Combination Class	Trial Number	Phase	Status
Pancreatic	RO7198457 (mRNA-based)	Atezolizumab, mFOLFIRINOX	Checkpoint inhibitor (PD-L1), chemotherapy	NCT04161755	I	Recruiting
Various lung cancers	NEO-PV-01 (peptide) + poly-ICLC	Pembrolizumab, Carboplatin, Pemetrexed	Checkpoint inhibitor (PD-1), chemotherapy	NCT03380871	I	Active, not recruiting
Glioblastoma	NeoVax (peptides + poly-ICLC)	RT, Pembrolizumab	RT, checkpoint inhibitor (PD-1)	NCT02287428	I	Active, not recruiting
Lymphocytic leukemia	NeoVax (peptides + poly-ICLC)	Cyclophosphamide	Chemotherapy	NCT03219450	I	Not yet recruiting
Glioblastoma	Peptide + poly-ICLC	Tumor treatment fields	Other	NCT03223103	I	Recruiting

*HCC* hepatocellular carcinoma, *NSCLC* non-small-cell lung carcinoma, *RCC* renal cell carcinoma, *RT* radiation therapy, *SCC* squamous cell carcinoma, *TNBC* triple-negative breast cancer

### Combination with checkpoint inhibitors

Checkpoint inhibitors targeting either the PD-1/programmed death-ligand 1 (PD-L1) axis or CTLA-4 reduce negative regulation of activated T cells and have been clinically approved for use in combination with the therapeutic cancer vaccine sipuleucel-T [36]. Additionally, multiple studies have reported that neoepitope-reactive T cells express high levels of PD-1 following treatment with monotherapy neoepitope vaccine, making checkpoint inhibitors an ideal candidate for combination therapy [10, 18, 28].

#### Preclinical studies

In mice bearing CT2A glioma, three reactive neoepitopes were identified that elicited immune responses prior to vaccination; these neoepitopes were chosen for combination therapy with anti-PD-L1. Overall, 60% of mice bearing CT2A tumors had long-term survival when treated with the neoepitope vaccine, consisting of 27-mer peptides and poly-ICLC, in combination with anti-PD-L1, compared to median overall survival of 17.5 and 25 days for mice treated with monotherapy vaccine or anti-PD-L1 alone, respectively. Additionally, compared to monotherapy treatments, combination therapy increased the number of tumor-infiltrating neoepitope-specific CD8 T cells [20].

In the MC38 murine colon carcinoma model, three studies investigated the use of a peptide vaccine, targeting a previously identified neoepitope in the Adpgk protein [14], delivered via synthetic nanoparticles [31, 32] or albumin/albumin-binding vaccine (AlbiVax) complexes [28]. These studies all showed that delivering peptides via nanovaccines induced immunity and decreased tumor progression, compared to non-treated mice or those treated with peptide-based vaccines; however, the tumors failed to regress [28, 31]. To improve efficacy, nanovaccines were combined with anti-PD-1, which resulted in robust neoepitope-specific cytotoxic T lymphocyte responses [31], extended survival times [32], and complete tumor regression in greater than 50% of treated mice [28, 31]. Responses were CD8 mediated [28, 32] and cured mice resisted rechallenge [28, 31]. Comparatively, mice treated with soluble Adpgk in combination with anti-PD-1 had a lower rate of tumor regression [28, 31]. In a similar study, nanodiscs were loaded with previously identified neoepitopes from the B16F10 murine melanoma model, as well as with an epitope from tyrosinase-related protein two, a melanoma-associated antigen. Mice bearing B16F10 tumors were treated with these multi-epitope nanodiscs in combination with anti-PD-L1 and anti-CTLA-4, resulting in cures in 90% of mice [31].

Another study investigated combining checkpoint inhibitors with neoepitope vaccines delivered in Great Ape–derived adenovirus (GAd) [30]. Taking advantage of this vector’s ability to encode for large antigens, seven previously identified MC38 neoepitopes [14] (each encoded by 25 amino acids) were joined to form a single gene and cloned into the GAd vector. Monotherapy vaccine was ineffective in mice bearing large tumors. However, combining the GAd neoepitope vaccine with either anti-PD-1 or anti-PD-L1 resulted in tumor regression in approximately 30% of mice. To further evaluate this platform, the GAd vector was designed to contain 31 neoepitopes identified in the CT26 murine colorectal carcinoma model. Monotherapy vaccine induced T-cell immunity in naïve mice, but only controlled tumor growth as a prophylactic vaccine or as an early intervention in a lung metastases model, not in large, established, subcutaneous tumors, despite the presence of vaccine-induced T cells within the tumor. However, combination with anti-PD-1 resulted in complete tumor regression in approximately 50% of mice. Responders were protected from rechallenge, indicating the development of a memory response. Additionally, responders demonstrated a potent immune response at the tumor site, characterized with higher frequencies of IFNγ^+^CD8 T cells, upregulation of genes in pathways relating to innate and adaptive immune activation, and diversification of intratumoral T-cell repertoire that was dominated by specific T-cell clones [30].

#### Clinical studies

**Fig. 2:**
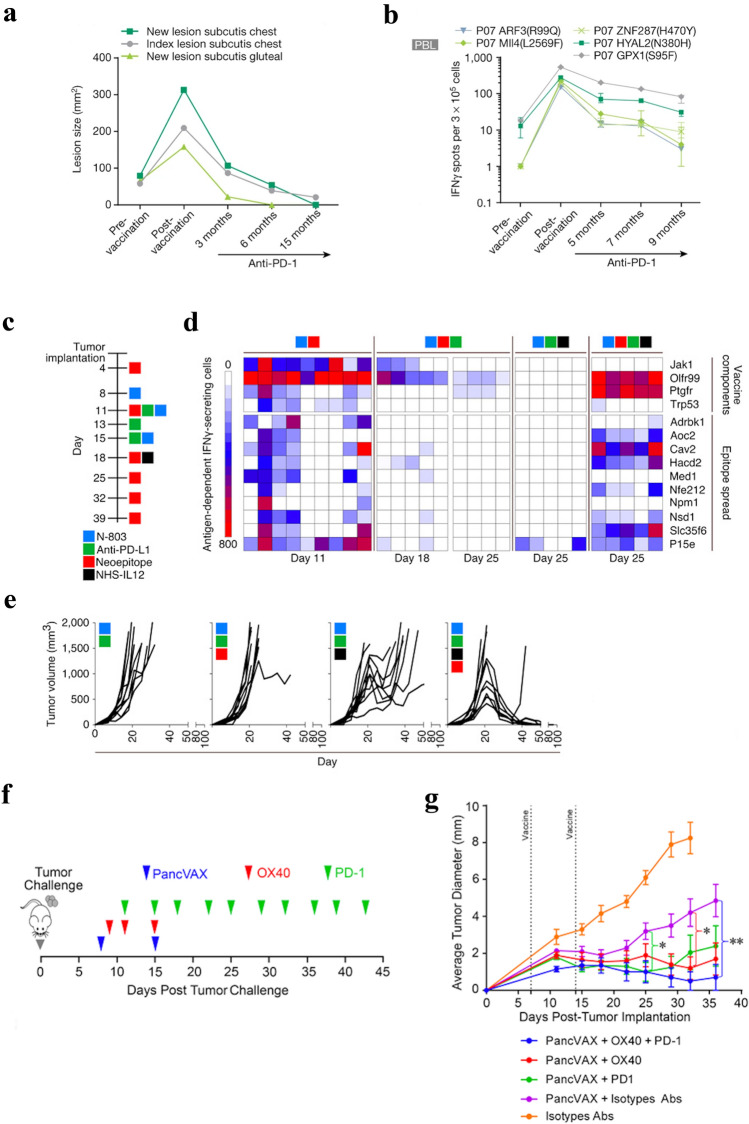
Neoepitope vaccine in combination with other cancer therapies leads to improved tumors response rates. **a** Computer tomography measurement of lesion size and (**b**) vaccine-induced neoepitope-specific ex vivo ELISPOT responses (measured in peripheral blood) of a stage IV melanoma patient receiving neoepitope vaccination, followed by anti-PD-1. Panels (a) and (b) reproduced with permission from [10]. Copyright (c) 2017, Springer Nature. **c** Treatment schedule of mice bearing MC38 colon carcinoma tumors. **d** IFNγ ELISPOT analysis on days 11, 18, and 25 post-tumor implantation against peptides contained within the vaccine (top) or MC38 neoepitopes not contained within the vaccine or p15e (bottom) of mice treated as in (c). **e** Tumor growth curves (*n* = 10) of mice treated according to the schedule in part (c). Panels (c), (d) and (e) reproduced with permission from [34]. Copyright (c) 2019, American Association for Cancer Research. **f** Treatment schedule of mice bearing Panc02 pancreatic cancer tumors. **g** Tumor growth curves of mice treated according to the schedule in part (f). Panels (f) and (g) reproduced with permission from [18]. Copyright (c) 2018, American Society for Clinical Investigation

Clinically, complete response following anti-PD-1 therapy in melanoma patients is typically less than 10%. However, two clinical studies have reported improved response rates following administration of pembrolizumab (anti-PD-1) to patients who developed recurrent disease or experienced relapse after administration of a neoepitope vaccine [9, 10]. Following surgical resection of high-risk melanoma, investigators evaluated a vaccine containing neoepitope long peptides, one tumor-associated antigen, and adjuvants. Six stage IIB/C or stage IVM1 a/b melanoma patients were evaluated; all patients generated de novo immune responses against neoepitopes following vaccination, as measured by ex vivo IFNγ ELISPOT of peripheral blood mononuclear cells. Response was measured by evidence of recurrence and patients who entered the study with stage IIIB/C disease (67%) had no disease recurrence at 25 months. However, patients who entered with stage IVM1b disease (33%) had recurrence at follow-up and received anti-PD-1. The addition of anti-PD-1, associated with the persistence and broadening of neoepitope-specific T-cell responses, revealed new CD4 and CD8 responses. After four doses of checkpoint inhibitor, complete radiographic response was achieved and sustained [9]. Another study also investigated personalized melanoma vaccines. Stage III and IV melanoma patients, in either complete remission, partial remission, or with stable disease, received an RNA-based poly-neoepitope vaccine containing 10 mutations (divided into two synthetic RNAs). After administration of a monotherapy vaccine, T cell responses were detected against 60% of the predicted neoepitopes, with all 13 patients developing T cells specific for at least three neoepitopes, one-third of which were detectable prior to vaccination. Patients with no radiologically detectable disease (62%) at the start of vaccination demonstrated robust immune responses and remained recurrence-free for the duration of the study. Additionally, two patients with measurable lesions at the start of vaccination developed vaccine-related objective clinical responses. One patient had a complete response of multiple progressing metastases following neoepitope vaccination. Another patient had rapid disease progression after neoepitope vaccination began, despite the generation of strong immune responses to six of 10 neoepitopes. To improve responses, this patient was given anti-PD-1 after stopping vaccination. After anti-PD-1 therapy, there was an 80% decrease in multiple lesions, which eventually led to a complete response (Fig. 2a) Interestingly, vaccine-induced T cells were still detectable up to 9 months following cessation of vaccination (Fig. 2b) [10]. Together these preclinical studies and preliminary data from clinical studies indicate the potential of combining neoepitope vaccine with checkpoint inhibitors. There are numerous studies currently under investigation combining neoepitope vaccine with various checkpoint inhibitors, including anti-CTLA-4 (NCT02950766), anti-PD-1 (NCT04266730, NCT02897765, NCT04024878, NCT03568058, NCT03633110), anti-PD-L1 (NCT03359239, NCT03289962, NCT03199040), and multiple checkpoint inhibitors (NCT03929029, NCT03422094, NCT03953235, NCT03639714, NCT03598816).

### Combination with cytokines

Cytokines have also been investigated for combination therapy with neoepitope vaccines and checkpoint inhibitors. As shown previously, neoepitope vaccines recruit reactive T cells to the tumor microenvironment and checkpoint inhibitors decrease immune suppression of T cells. The addition of cytokines to this combination expands and activates immune cells in the tumor microenvironment. Two cytokines under investigation for combination therapy are IL-15 and IL-12. IL-15 promotes the expansion of T cells and natural killer (NK) cells, without expanding the regulatory T cell (Treg) compartment [37]. Similarly, IL-12 activates and expands NK and T cells and promotes the production of IFNγ, which in turn stimulates more facets of the anti-cancer immune response. Various preclinical studies have shown that antitumor activity of IL-12 monotherapy can be improved by combination with TAA vaccines [38].

In the MC38 murine colon carcinoma model, four immunogenic neoepitopes were identified and delivered as 9-mer peptides in Montanide ISA 51 VG or via adenoviral vector. Treatment with monotherapy peptide vaccine did not provide any survival benefit. However, when administered with anti-PD-L1 and N-803, an IL-15 superagonist, an increase in immunogenicity of the vaccine was observed, which correlated to an extension in survival. To further improve efficacy, the tumor-targeted immunocytokine NHS-IL12 was added to the treatment regimen. This quadruplet therapy led to tumor regression in 60% of mice and cured mice resisted rechallenge. Mice treated with quadruplet therapy exhibited immunity against vaccine components, but also against other MC38 neoepitopes expressed by the tumor, yet not contained within the vaccine, thereby indicating neoepitope spreading (Fig. 2c-e). Additionally, mice treated with neoepitope vaccine, N-803, anti-PD-L1, and NHS-IL12 demonstrated increased CD8 T-cell infiltration and expansion of gene transcripts relating to the innate immune system and T-cell activation and effector function [34].

Another cytokine under consideration for combination therapy is Flt3 ligand, which expands DC and NK populations, and induces maturation of T, B, and NK cells. Like IL-12, Flt3 ligand reduced tumor growth and induced immune-cell infiltration in preclinical models as a monotherapy and enhanced therapeutic efficacy of cancer vaccines targeting TAAs [39]. Clinically, IL-12 and recombinant Flt3 ligand are under investigation for combination with neoepitope vaccine (NCT04015700, NCT04251117, NCT02129075).

### Combination with immune-stimulatory molecules

Combination therapies have also investigated utilizing other immune-stimulatory molecules, including costimulatory molecules and synthetic compounds [18]. Costimulatory molecules, such as OX40 and CD40, are expressed on T cells [40] or APCs, respectively [41], promoting the generation/expansion of adaptive immunity. Preclinically, 12 neoepitopes from the pancreatic cancer cell line Panc02 were identified and synthesized as 20-mer peptides and combined with a STING-based adjuvant and AddaVax (a squalene-based oil-in-water adjuvant) (PanVAX). Monotherapy vaccination led to decreased tumor growth rate until day 20, after which tumors progressed. Analysis of tumor infiltrating lymphocytes (TIL) revealed high expression of the exhaustion markers PD-1, LAG-3, Tim3, and Tbet. To counteract this exhaustion, the vaccine was combined with an anti-PD-1 and/or an OX40 agonist antibody [18]. TIL isolated from mice treated with vaccine plus anti-PD-1 expressed low levels of IFNγ and high surface expression of PD-1, and had increased frequencies of Tregs; TIL isolated from mice treated with vaccine plus anti-OX40 produced high levels of IFNγ, and had low PD-1 expression and decreased frequencies of Tregs. In comparison, TIL from mice treated with triplet therapy had decreased expression of exhaustion markers compared to either doublet therapy. Overall, this indicated that vaccine and OX40 agonist antibody induced a strong immune response at the tumor site and combination with anti-PD-1 further decreased exhaustion markers. This synergistic anti-tumor effect correlated with improved survival. Vaccine combined with anti-PD-1 or anti-OX40 resulted in complete tumor eradication in 60% or 70% of mice, respectively. However, mice treated with triplet therapy exhibited 90% tumor eradication (Fig. 2f, g), with 80% of cured mice resisting rechallenge. Importantly, mice that resisted rechallenge exhibited robust T-cell responses toward at least one neoepitope, while the mouse that failed to reject rechallenge had limited response to any of the neoepitopes contained within the vaccine, indicating a vaccine-specific anti-tumor response [18].

The costimulatory molecule CD40 is also being investigated for combination therapies. Clinically, a CD40 monoclonal antibody (mAb) as a monotherapy resulted in minimal response rates. However, increased levels of systemic IL-12, and increased APC and T-cell activation were reported following administration, indicating the potential for combining with neoepitope vaccines [42].

Other immune-stimulatory agents include imiquimod and lenalidomide, which are synthetic compounds that stimulate the innate and adaptive immune responses. When used in combination with therapeutic cancer vaccines, both agents induced the enhanced generation of vaccine-specific cellular and humoral immune responses [43, 44], indicating that there is a strong rationale for combining these agents with therapeutic neoepitope vaccines; these combinations are currently being assessed clinically (NCT03597282, NCT02600949, NCT02721043).

### Combination with other immuno-oncology agents

Other immuno-oncology agents under consideration for combination with neoepitope vaccine include tumor-targeting mAbs and vaccines targeting TAAs. Rituximab, an anti-CD20 antibody used for B-cell malignancies, triggers cell death via signaling induced cell death, complement-mediated cytotoxicity, or antibody-dependent cellular toxicity [45]. It is currently under investigation in combination with neoepitope vaccines for the treatment of follicular lymphoma (NCT03361852).

Unlike neoepitope vaccines, TAA-targeting vaccines are off the shelf, making them more readily available, and more cost-effective to produce. In preclinical studies, a vaccine targeting TAAs and neoepitopes significantly inhibited tumor growth compared to a vaccine that only targeted one class of antigens [31], while a TAA-targeting vaccine in combination with other immuno-oncology agents induced epitope spreading toward neoepitopes [34]. Additionally, a clinical study reported increased immune responses in patients who received a vaccine targeting neoepitopes and a TAA versus patients who received a vaccine targeting only neoepitopes [10]. Thus, combining neoepitope-targeting vaccines with TAA-targeting vaccines allows for quicker vaccination, improved targeting of tumor heterogeneity, and the potential for epitope spreading. Ongoing clinical studies are investigating combining neoepitope vaccines with vaccines targeting TAAs (NCT03532217, NCT02129075).

### Combination with “non-immune” agents

In addition to immuno-oncology agents, radiation therapy and chemotherapy are also candidates for combination therapy. Radiation eradicates tumor cells via direct and indirect DNA damage, leading to cell death. However, recent research has demonstrated that local irradiation can also cause systemic responses (the “abscopal effect”). This is due to the ability of radiation to elicit tumor-specific T cells at the tumor site, which then traffic systemically. This ability to function as an in situ vaccine makes radiation therapy a rational possibility for combination with neoepitope vaccine. Indeed, preclinical studies have demonstrated that TAA-targeting vaccines combined with radiation leads to tumor regression, development of immunological memory, and long survival times [26, 46]. Preclinical work has also shown the potential of combining neoepitope vaccines with radiation therapy. In a CT26 colorectal carcinoma lacking gp70, an immunodominant epitope derived from an endogenous retrovirus (CT26-gp70KO), investigators aimed to induce epitope spreading toward a neoepitope not contained within the vaccine (Smc3) [35]. Mice bearing CT26-gp70KO tumors were treated with RNA delivered via lipoplex, containing five previously identified MHC class II neoepitopes [19], with or without 14 Gy of local irradiation. Neither vaccine nor radiation alone induced Smc3-specific CD8 T cells. However, the combination of the two therapies induced high levels of antigen-specific IFNγ production, indicating the ability of this combination to induce epitope spreading at the tumor site [35].

Similarly, chemotherapeutic agents can have immunomodulatory effects, in addition to their direct cytotoxicity to cancer cells. The different classes of chemotherapeutics and their contribution to immune modulation have previously been reviewed [26], highlighting their potential for combination with neoepitope vaccines. Additionally, chemotherapy has already been successfully combined with TAA-targeting vaccines in preclinical models and clinical studies and resulted in an increase in antigen-specific T cells and improved survival [26]. The ability of radiation therapy and chemotherapy to directly kill tumor cells, thereby releasing additional antigens for T-cell targeting, along with their immunomodulatory properties, makes both therapies strong candidates for combination with neoepitope vaccines. Current studies combining neoepitope vaccines with radiation (NCT02287428) or chemotherapy (NCT04161755, NCT03380871, NCT03219450) are ongoing.

## Conclusions and outlook

Reports from initial clinical studies utilizing vaccines to target neoepitopes reveal the potential for developing personalized immunotherapies. However, despite the induction of strong immunity following vaccination, efficacy was not always observed. Therefore, preclinical work has now focused on developing combination therapies to be used with neoepitope vaccines and data suggest that these combinations improve responses versus monotherapy vaccine alone. Combination therapy increased neoepitope-reactive T cells, induced epitope spreading to non-vaccine components, slowed tumor growth, and extended survival. Additionally, preclinical studies demonstrated that different methods of neoepitope delivery, such as the addition of alternate adjuvants or nanoparticle/nanovaccine or adenoviral delivery can further enhance immunogenicity. Indeed, initial reports from clinical trials combining checkpoint inhibitors with neoepitope vaccines are promising, with some patients developing sustained responses. Next generation combination therapies in ongoing clinical trials aim to build on preclinical successes and expand the repertoire of potential cancer therapies that can be combined with neoepitope vaccines. As research moves toward clinical translation, these combination therapies will require rational selection of agents and order of administration. These include multiple checkpoint inhibitors, costimulatory agonist antibodies, tumor-targeting mAbs, cytokines, TAA-targeting vaccines, and standard-of-care radiation or chemotherapy. The potential for these combinations is encouraging, as they have been successfully combined with TAA-targeting vaccines. Moving forward, results from these clinical trials will guide the field and inform on which combinations hold the most potential for improving overall clinical efficacies.

## Abbreviations

AlbiVax: Albumin/albumin-binding vaccine
APC: Antigen-presenting cell
DC: Dendritic cell
EGFRvIII: Epidermal growth factor receptor class III variant
GAd: Great Ape–derived adenovirus
GM-CSF: Granulocyte macrophage-colony stimulating factor
IDH1: Mutant isocitrate dehydrogenase 1
NGS: Next generation sequencing
mAb: Monoclonal antibody
NK: Natural killer
PD-1: Programmed death-1
PD-L1: Programmed death-ligand 1
TAA: Tumor-associated antigen
TIL: Tumor-infiltrating lymphocytes
TNF: Tumor necrosis factor
T-VEC: Talimogene laherparepvec
